# Supplementary material for: Assessing the biogeography of marine giant viruses in four oceanic transects
Source: ISME Commun. 2023 Apr 29;3:43. doi: 10.1038/s43705-023-00252-6 (PMC10148842; doi:10.1038/s43705-023-00252-6)

Figure S1. Phylogeny of all giant viruses used for metagenomic mapping. The order-level classification of *Nucleocytoviricota* viruses is denoted by the branch colors and color strip. The genomes with mapping to transect metagenomic data are denoted on the outermost bar plot, with the height of the bar corresponding to average abundance (RPKM). The six most widespread genomes are marked with red circles.

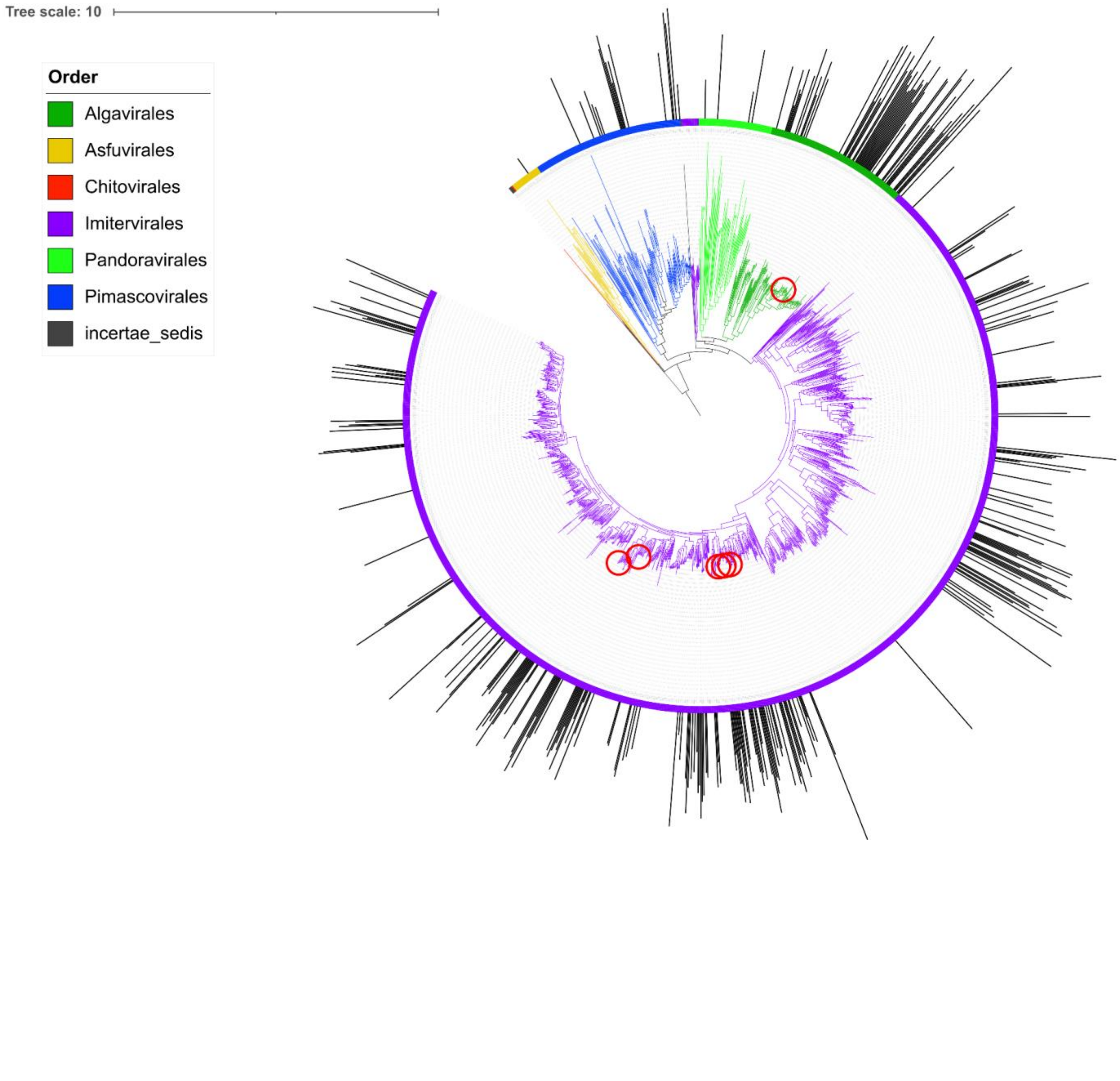

Figure S2. Summary of the taxonomy of detected giant viruses. The area of each rectangle is proportional to the number of identified viral genomes in the respective taxon.

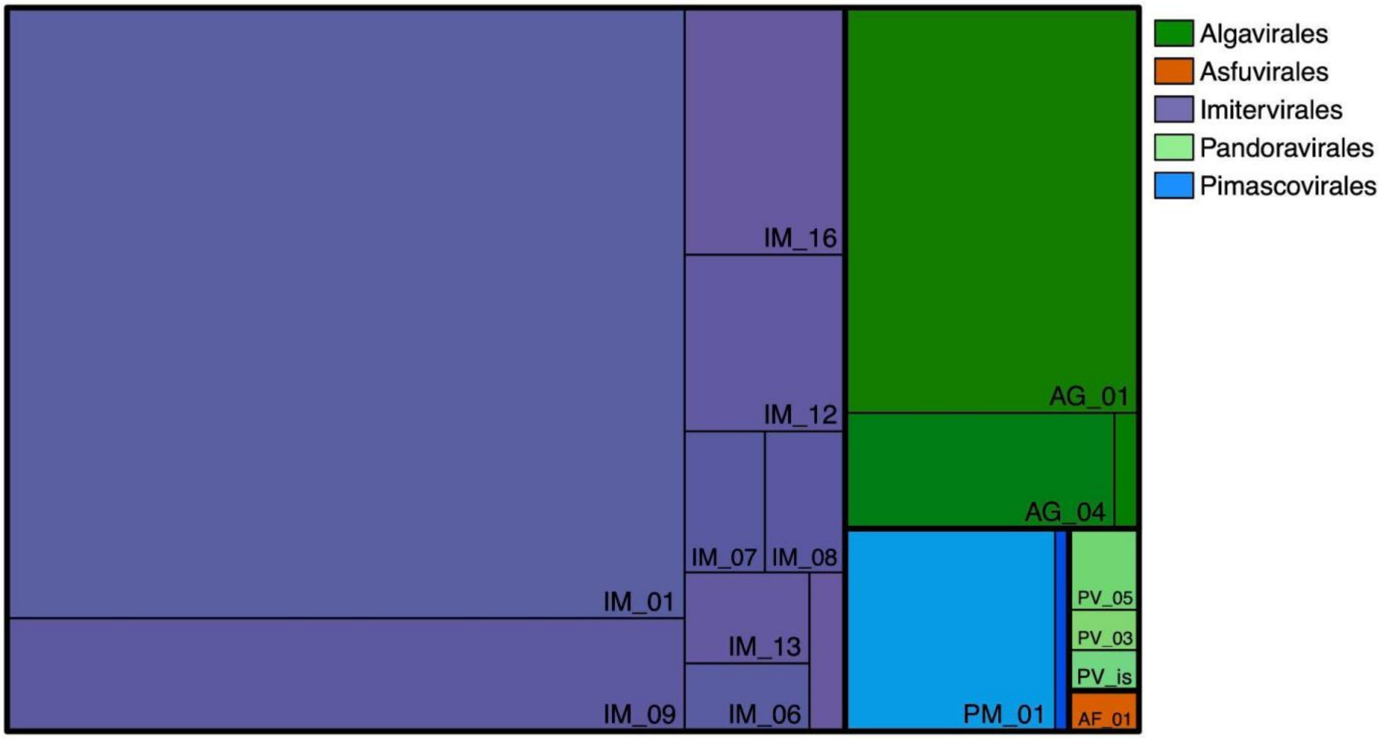

Figure S3. Geographic distribution of the five *Mesomimiviridae* viruses and one *Prasinoviridae* virus that were widespread in oligotrophic waters. The size of the bubbles is scaled to the abundance of the virus at a given location. The color of the bubbles shows the taxonomic order of the genome (purple: *Imitervirales*, green: *Algavirales*).

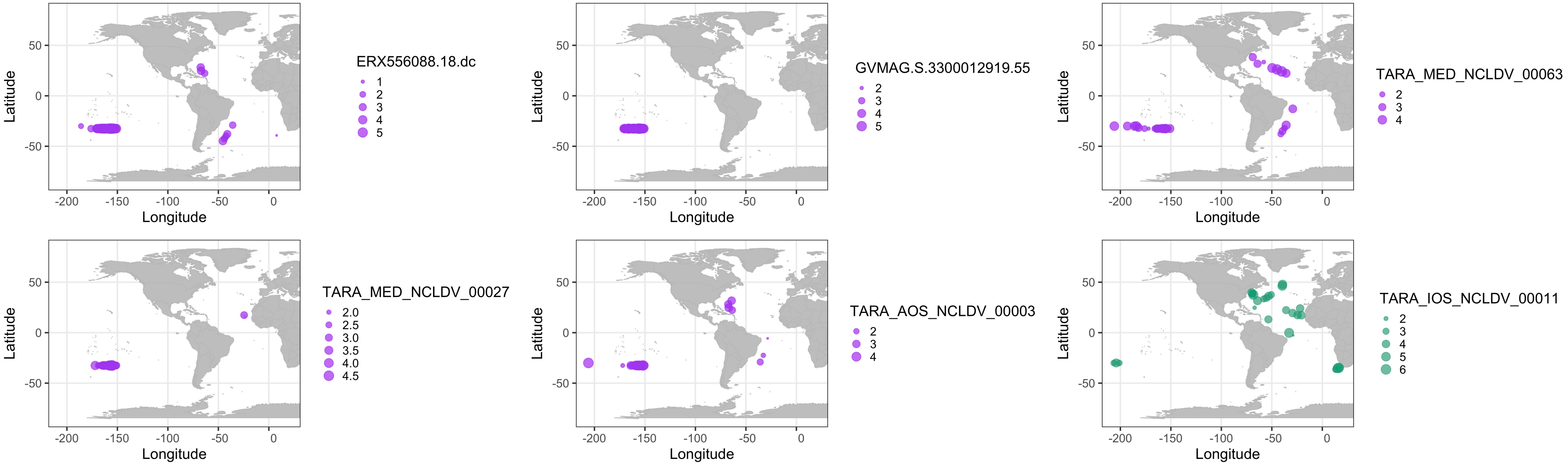
ERX556088.18.dc
1
2
3
4
5
GVMAG.S.3300012919.55
2
3
4
5
TARA_MED_NCLDV_00063
2
3
4
TARA_MED_NCLDV_00027
2.0
2.5
3.0
3.5
4.0
4.5
TARA_AOS_NCLDV_00003
2
3
4
TARA_IOS_NCLDV_00011
2
3
4
5
6
Latitude
Longitude
50
0
-50
-200
-150
-100
-50
0

Figure S4. Distribution of viruses of the *Mirusviricota,* and the order *Pimascovirales* and *Pandoravirales* throughout the water column along the transects, showing the viral abundance (calculated in log RPKM) of (A) *Mirusviricota* viruses (B) viruses of the *Pimascovirales* order (C) viruses of the *Pandoravirales* order. Samples were ordered based on the distance along transects, beginning from the first sampling location of cruise tracks (0 km). White dots denote the sampling location along the transect of each sample.

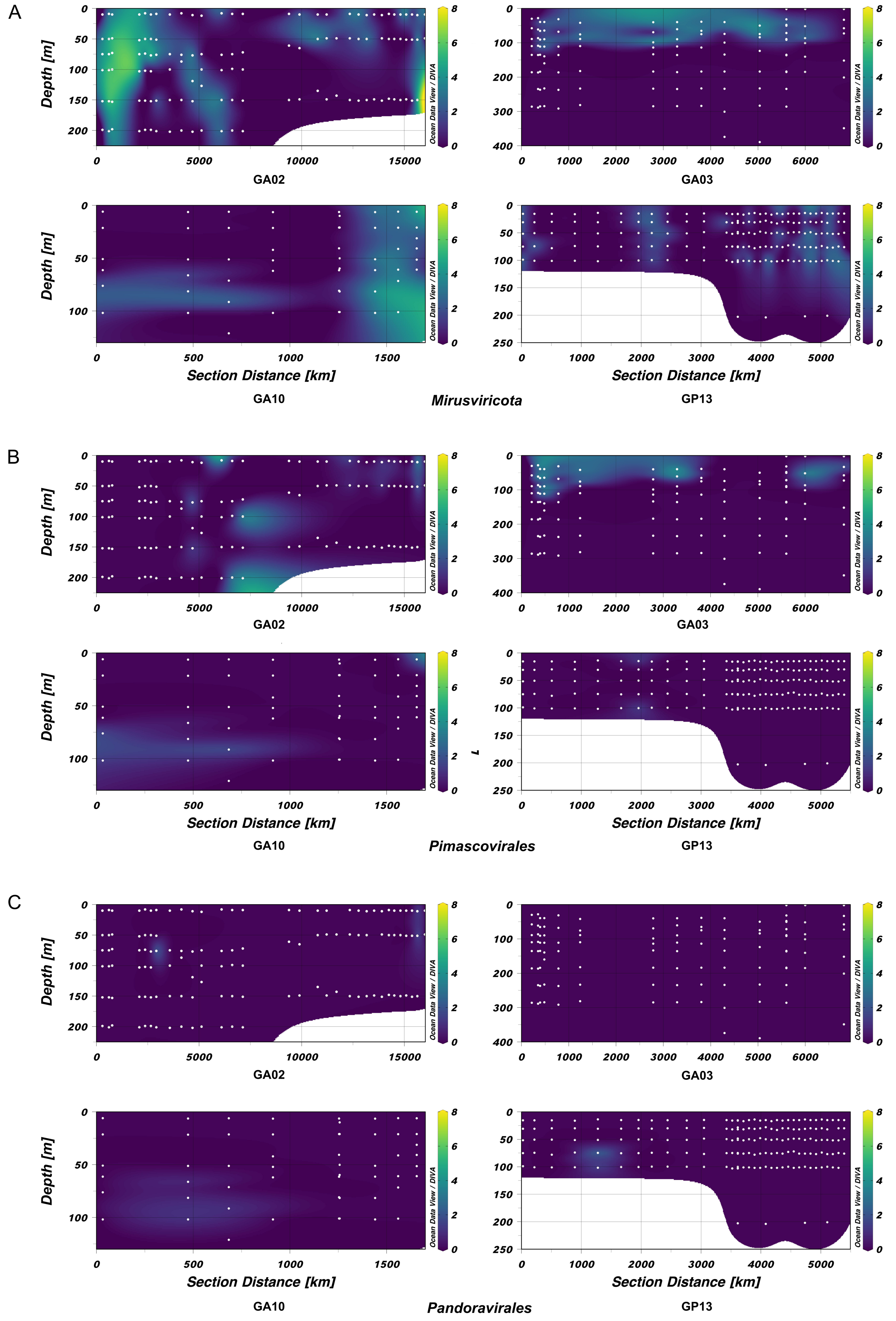
A
B
C
Depth [m]
Section Distance [km]
Ocean Data View / DIVA
GA02
GA03
GA10
GP13
Mirusviricota
Pimascovirales
Pandoravirales

Figure S5. Comparison of overall giant virus richness between three depth ranges in the water column across all transects. Stars showing significant difference between groups (Wilcox test, p-values < 0.05) (* < 0.05, ** < 0.01, *** < 0.001, **** < 0.0001)

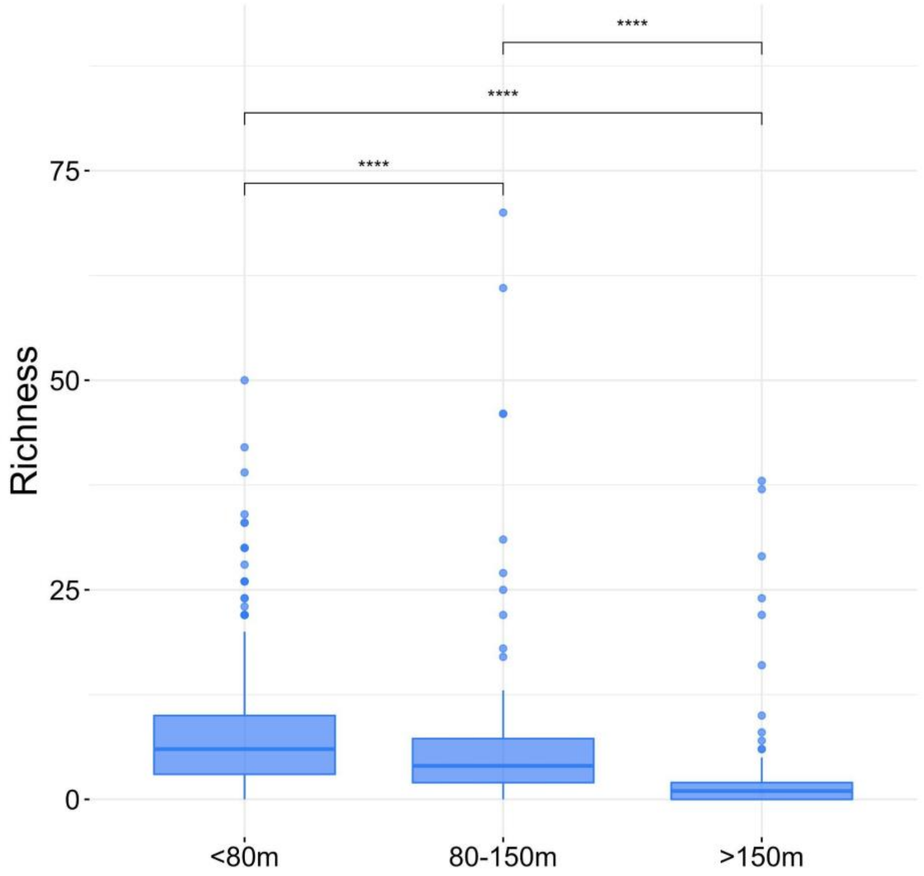

Figure S6. Latitudinal pattern of giant viral diversity across TARA Oceans samples. Stars showing significant difference between two latitudinal groups (Wilcox test, p-values < 0.05) (* < 0.05, ** < 0.01, *** < 0.001, **** < 0.0001) Panels left: Shannon's Index; right: Community richness. EQ, Equator.

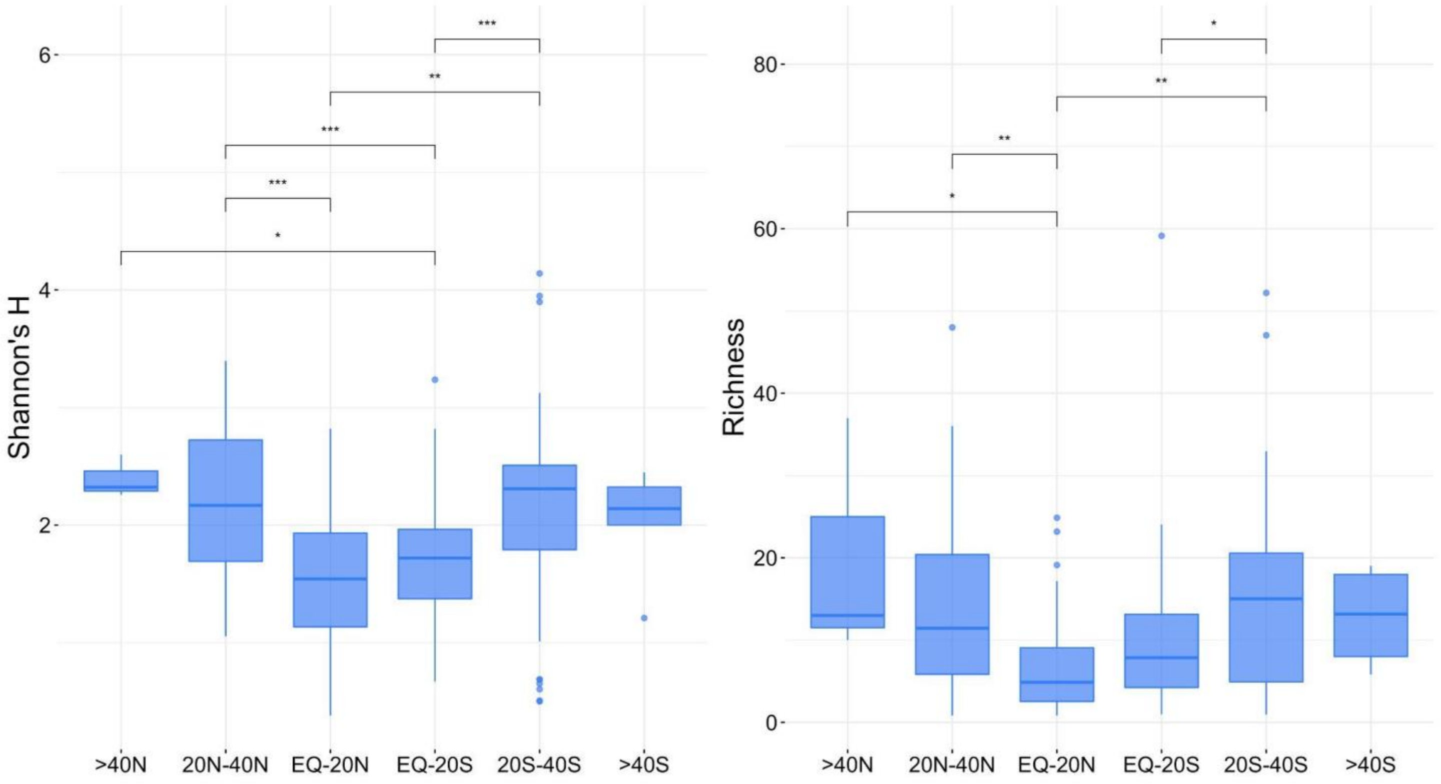

Figure S7. Unique genomes and genomes shared between the three latitudinal zones. Horizontal bars (right) indicate the total number of genomes found in each zone; red dots show the average size of all genomes found in each zone. Black dots indicate the presence in one or multiple zones; the corresponding vertical bars indicates the number of genomes with the presence described by the dots.

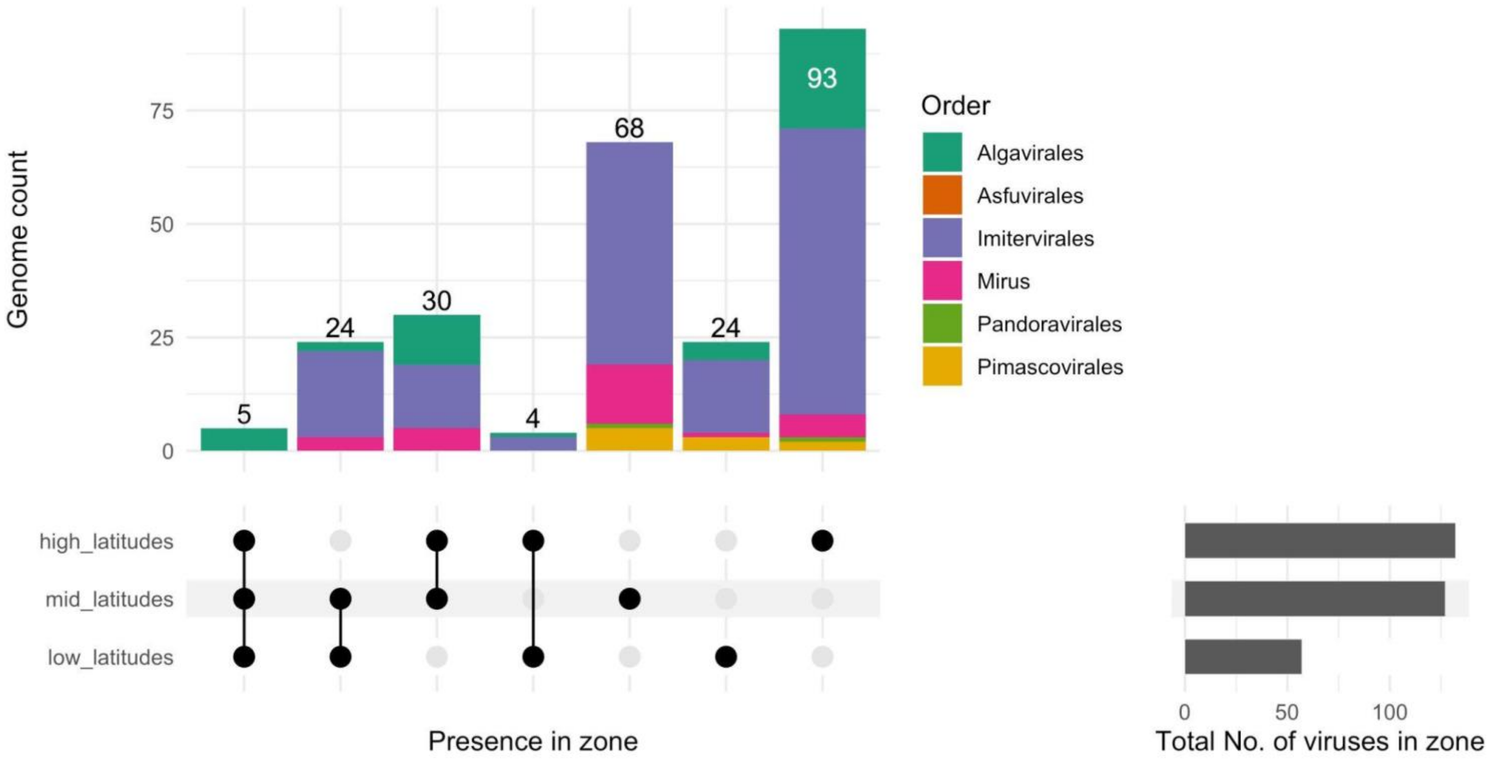

Supplement: Supplementary file 1 — Supplementary Figures [file 43705_2023_252_MOESM1_ESM.pdf]
